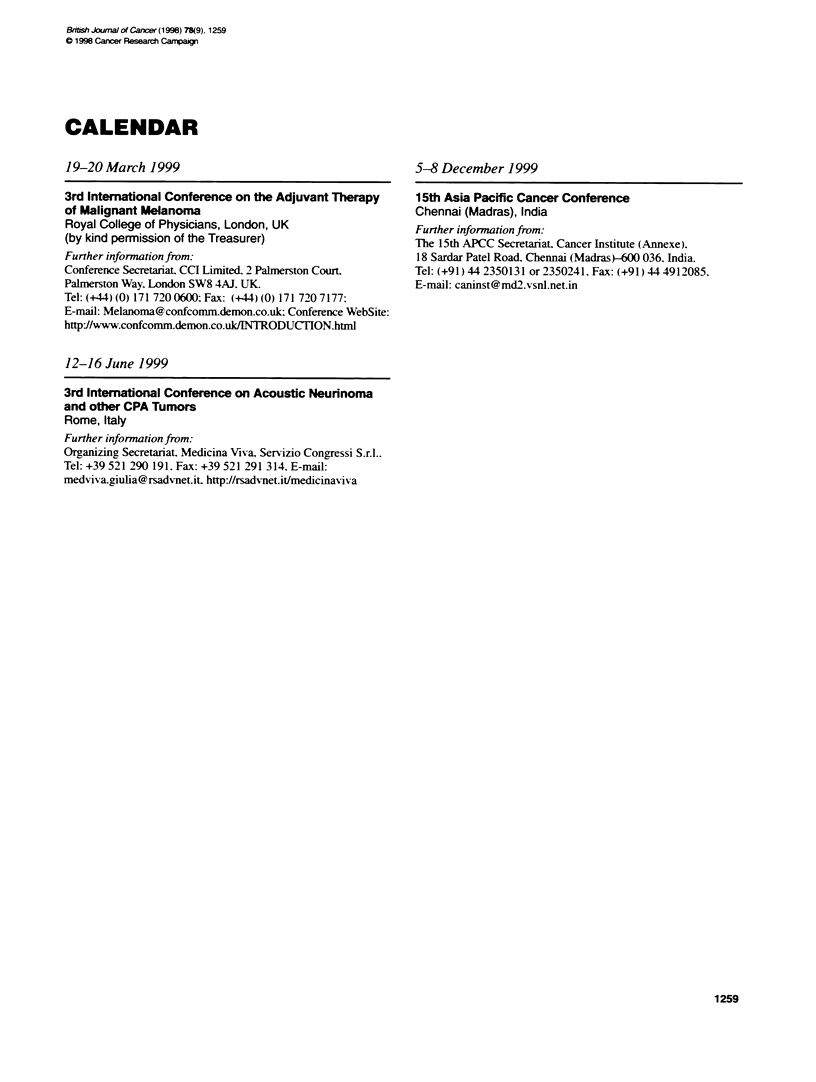# Calendar

**Published:** 1998-11

**Authors:** 


British Journal of Cancer (1998) 78(9), 1259

© 1998 Cancer Research Campaign

### CALENDAR

*19–20 March 1999*

**3rd International Conference on the Adjuvant Therapy of Malignant Melanoma**
Royal College of Physicians, London, UK
(by kind permission of the Treasurer)

*Further information from:*
Conference Secretariat, CCI Limited, 2 Palmerston Court, Palmerston Way, London SW8 4AJ, UK.
Tel: (+44) (0) 171 720 0600; Fax: (+44) (0) 171 720 7177;
E-mail: Melanoma@confcomm.demon.co.uk; Conference WebSite: http://www.confcomm.demon.co.uk/INTRODUCTION.html

*12–16 June 1999*

**3rd International Conference on Acoustic Neurinoma and other CPA Tumors**
Rome, Italy

*Further information from:*
Organizing Secretariat, Medicina Viva, Servizio Congressi S.r.l.,
Tel: +39 521 290 191, Fax: +39 521 291 314, E-mail: medviva.giulia@rsadvnet.it, http://rsadvnet.it/medicinaviva

*5–8 December 1999*

**15th Asia Pacific Cancer Conference**
Chennai (Madras), India

*Further information from:*
The 15th APCC Secretariat, Cancer Institute (Annexe), 18 Sardar Patel Road, Chennai (Madras)–600 036, India.
Tel: (+91) 44 2350131 or 2350241, Fax: (+91) 44 4912085,
E-mail: caninst@md2.vsnl.net.in